# Supplementary material for: Implementation of Virtual Communities of Practice in Healthcare to Improve Capability and Capacity: A 10-Year Scoping Review
Source: Int J Environ Res Public Health. 2022 Jun 29;19(13):7994. doi: 10.3390/ijerph19137994 (PMC9265616; doi:10.3390/ijerph19137994)
Supplement: Supplementary file 1 [file ijerph-19-07994-s001.zip › Table S1_Table of study characteristics_reviews.pdf]

**Table S1:** Table of characteristics for review papers

| | *Lead Author, year (country published)* | *Review type* | *Studies reviewed* | *Context* | *Aim* | *Outcome measures* | *Key findings* |
|---|---|---|---|---|---|---|---|
| **1** | Adedoyin, 2016 (United States) | Rapid Review | 10 | Social work | To examine the literature for studies on VCoP's in social work | Digital tool used, outcomes, environment, design | -Online tools are being used more frequently in social work.<br>-Students accept more readily than lecturers and supervisors<br>-Cautious of privacy and ethical issues<br>-Best practice and continued learning through online platforms should be encouraged. |
| **2** | Barnett et al, 2012 (Australia) | Literature Review | 22 | GP training | To explore if there is any evidence of VCoPs for GP training + to identify evidence-based guidelines for developing a VCoP | Support or negative count in the literature for 6 key themes for developing a VCoP | No studies specific for GP training.<br><br>6 key themes for developing a VCoP (Probst and Borzillo) and support or negative count from the literature: Leadership (support:18, negative: 4), Objectives (support:15, negative:3), Sponsorship (support:12, negative: 0), Boundary Spanning (support: 12, negative:3), Risk-free environment (support: 6, negative:2) and measurements (support:6, negative:0).<br>Two additional themes from the authors: technology (support:16, negative:7), community (support: 19, negative:2)<br>Further research is needed to validate the proposed framework. |
| **3** | Mc Loughlin et al, 2018 (Ireland and United Kingdom) | Integrated Review | 19 articles | Health and social care professionals | To find uses for supporting interprofessional education (IPE) and interprofessional collaboration (IPC) activities and determine strengths and weaknesses | Definitions and approaches technological infrastructure reported benefits participation issues trust and privacy and technical ability | "..there is some evidence that VCoPs can offer an informal method of professional and interprofessional development for clinicians, and can decrease social and professional isolation. However, for VCoPs to be successful, issues of privacy, trust, encouragement and technology need to be addressed" |
| **4** | Ranmuthugala et al, 2011 (Australia) | Systematic Review | 31 primary research, 2 systematic reviews | Healthcare | Review of how/why VCOP's are developed and if they assist in healthcare | Composition and purpose of CoP's in healthcare, means of interaction, methods of knowledge exchange, improving healthcare performance | -UK most common country of origin<br>-most of the CoPs described included more than one profession/organisation<br>- difficult to compare communication methods due to differences in reporting<br>-16/31 studies reported meeting face-to-face at least once<br>-24/31 used qualitative methods<br>-most studies used data from a single approach to gather data (e.g., interviews or observations)<br>-Require a flexible framework aiming to "guide" rather than "prescribe" |
| **5** | Rolls et al, 2016 (Australia, United Kingdom, United States) | Integrative literature review | 72 | Healthcare professionals | Review the use of social media by healthcare workers. | Research methods and study quality, use of social media by health care professionals, Web-based posting behaviours and | -Evaluation of 3 studies suggests that social media use is mediated by a positive attitude and accessibility<br>-Most commonly established to create a forum where relevant specialty knowledge and professional issues could be shared/discussed (n=17).<br>-More frequent reading/accessing behaviour, low posting behaviours by most |

| | | | |
|---|---|---|---|
| | | mediating factors of Web-based posting | -The most common Web-based activity was request for/supply of specialty-specific clinical information. Facilitated by a Web-based culture of collectivism, reciprocity, and a respectful non-competitive environment.<br>-Findings suggest that healthcare professionals view virtual communities as enabling them to make more informed practice decisions<br>-Tribal behaviours may limit knowledge sharing<br>-Further research required to assess if it improves patient outcomes |
