# Supplementary material for: Implementation of Virtual Communities of Practice in Healthcare to Improve Capability and Capacity: A 10-Year Scoping Review
Source: Int J Environ Res Public Health. 2022 Jun 29;19(13):7994. doi: 10.3390/ijerph19137994 (PMC9265616; doi:10.3390/ijerph19137994)
Supplement: Supplementary file 1 [file ijerph-19-07994-s001.zip › Table S2_Table of study characteristics_case reports.pdf]

Table S2. Table of study characteristics case reports

	Lead Author, year (Country)	*Context*	*Health professionals involved*	*Size of the network*	*Technological Infrastructure*	*Aim*	*Outcome measures*	*Key findings*
**1**	Abos Mendizabal et al, 2013 (Spain)	Primary Care	Primary care professionals	1627 registered	IdeaScale Software	To assess development, implementation, take-up, participation, use and opinions of a VCoP	Use of the VCoP	-Satisfactory participation and acceptance (90 actively participated)<br>-Confirms framework by Probst and Borzillo that essential criteria are leaders and facilitators, a wide base of potential participants, clear objectives, trust, a user-friendly platform, transparency and feedback about ideas<br>Facilitator role: coordinates 3 support groups, ideas innovation and management to ensure progress of ideas<br>New initiatives + increase role of health professionals to ensure sustainability
**2**	Alary Gauvreau et al, 2019 (Canada)	Aphasia Rehabilitation	Speech language pathologists	13	Adobe Connect	Describe and evaluate VCoP for aphasia rehabilitation	Logbooks and observation: logic and thematic analysis 5 educational aims: 1. knowledge acquisition 2. social interaction 3. knowledge sharing; 4. knowledge creation; 5. identity building"<br><br>"Level of participant engagement, the material shared and created by participants, the factors influencing the participants' appreciation of the activities of the CoP, and the impacts of participant engagement".	Resulted in more participation than anticipated.<br>Positive: All participants found the VCoP taught them about clinical tools & most reflected on their practice.<br>Negative: developed by researchers and therefore not driven by member needs (although positive results show it did address some member needs)
**3**	Coleman, 2012 (South Africa)	Doctors in rural hospitals	Doctors	10	N/A	Assess how knowledge is disseminated, to propose a VCoP framework for rural doctors to share with urban doctors.	Semi-structured open - ended interviews about ICT Infrastructure availability, doctors' workload, methods and frequency of consultation, task type and satisfaction of using a computer-mediated tool	New VCoP framework that is based on the following: responsibility, connectedness and reverence to support doctors in rural hospitals

**4**	Farrell et al, 2014 (United States)	Cancer Control	Cancer researchers and practitioners	"As of 2013 R2R had more than 1700 registered members"	Web-based dissemination program (P.L.A.N.E.T)	Describe how a VCoP for cancer control research can be put together and how it can help	Average comments generated in response to different discussion types, R2F NCI: Professional development, personal stories, soliciting of general response, posting of information or resources, self-promotion, or other promotion, sharing experiences, soliciting of information<br>Note: R2R used contact us feature and regular member surveys	-The most common cancer-issue posts were about: communication, dissemination and project implementation.<br>-Members most likely to respond when colleagues asking questions<br>-R2R used 3 main concepts: activity, content, interaction.<br>-Further evaluation required
**5**	Landes et al, 2019 (United States)	Psychotherapy	Department of Veteran Affairs, Dialectical Behaviour Therapy	"In the first 30 days, the site had 1,334 unique users. In the following months, it had between 24 and 684 unique users per 30 days"	Sharepoint web application developed by Microsoft	To describe the creation of a VCoP to support veteran affair providers to implement dialectical behaviour therapy (DBT) and therefore to assist other groups in implementing evidence-based therapy.	Web analytics	-Linked providers nationally and enabled discussions and a space to share information and resources.<br>-Also provided a link to other providers that use DBT.<br>-The VCoP met all the 7 characteristics of a VCoP:" facilitation, champion and support, objectives and goals, a broad church, supportive environment, measurement and feedback, technology and community" (Barnett et al)<br>-Involvement of technology and content experts
**6**	Lewis et al, 2018 (United States)	Dermatology	Two general dermatologists, two pediatric dermatologists, a dermatopathologist, a clinical psychologist and an advanced practice nurse meet with primary care providers (PCPs)	7 providers meet with 7-15 PCPs once per week.	Dermatology ECHO (Zoom) and ECHO tele Missouri Dermatology Image database mobile app	To demonstrate the dermatology ECHO approach via live interactive video technologies	Diagnosis of the case presentation and demonstration of the uses of dermatology ECHO	-An example of the benefits of dermatology ECHO<br>-Engages multidisciplinary team<br>-Reaches patients that would otherwise not get the care they require<br>-ECHO discussion of specific patient that resulted in improvement of condition<br>-Other sites also gained from the discussion

**7**	Murad et al, 2019 (Australia)	General Practitioners	Practicing GPs	7 continued for full course	The CFC Learning Hub<br><br>("The CFC Learning Hub is a secure, Web-based prototype HVCoP website, created using the Igloo Web-based social media software platform")	Development, description, and evaluation (particularly of method of testing engagement features) of an online case-based learning hub to help GP's understanding of osteoporosis.	Measurement of sessions and engagement (passive or active)	Key features:<br>-material was focused on clinical cases<br>-learning activities were interactive<br>-GPs were interested in practice-based web-based learning.<br>-GPs were more interested in topics regarding clinical application<br>Topics that were most engaging were: "about osteoporosis" and "prevention" were the most engaging topics and "shared decision making" was least active.
8	Sims, 2018 (United Kingdom)	Medical support for expeditions and people in extreme environments	Specialised medicine doctors	States 'over 500 members'	Website, email and blog.	Not specifically stated. 'The case study community is designed ot address the complex needs of communication, knowledge sharing and social inclusion'.	Researcher developed questionnaire with open questions built around themes derived from literature.	-services provided (website, email, blog) led to the development of a community.<br>-there is social cohesion and collaboration.<br>-the community has a distinct identity.<br>-the community facilitates greater effectiveness, quality and consistency of practice.<br>-members acquired, shared and retained knowledge.
**9**	Ting et al, 2018 (Canada)	Emergency Medicine	Canadian Emergency Medicine practitioners	N people not stated. 'From the website launch (January 1, 2016) through September 23, 2018, CanadiEM received > 2.5 million page views (now averaging > 120,000 page views/ month). CRACKCast podcasts downloaded > 750,000 times (>35,000 downloads/month). Website viewers hailed from 217 countries.	CanadiEM (2 websites and a podcast)	To develop a VCoP for collaboration, learning and knowledge dissemination	Website content & audience, education scholarship	Successful in developing a platform for knowledge dissemination<br>Had more than 2.5 million page views from 217 countries. Podcast downloaded 750 000 times. 782 published articles of which 71 underwent coached peer review process.<br>- Identity developed that values national collaboration on high-quality scholarship.
**9**	Wolbrink et al, 2017 (United States)	Pediatric critical care	Critical care clinicians	"OPENPediatrics has acquired over 7,443 registered users from over 1,800 hospitals in	OPENPediatrics6 (online social learning platform)	To describe and evaluate the online paediatric critical care CoP	Web-based surveys and site analytics	"From March 2013–November 2015, 28 WSPs [world shared practice videos] were launched on a variety of topics. They were viewed over 18,414 times by 1,864 viewers in 132 countries and 760 hospitals; 1,155 comments were

	132 countries as of November 2015"	(through face-to-face conferences and online)		submitted. Attending physicians/consultants were the largest audience (36% [671/1,864]), and 37% (30/81) of responding viewers that commented in WSPs watched in small groups. The WSP series was reported to add value to respondents' learning or teaching and to have had a positive impact on their knowledge or practice."
