# Supplementary material for: Implementation of Virtual Communities of Practice in Healthcare to Improve Capability and Capacity: A 10-Year Scoping Review
Source: Int J Environ Res Public Health. 2022 Jun 29;19(13):7994. doi: 10.3390/ijerph19137994 (PMC9265616; doi:10.3390/ijerph19137994)
Supplement: Supplementary file 1 [file ijerph-19-07994-s001.zip › Table S3_Table of Study characteristics_Other types of study.pdf]

Table S3. Table of study characteristics: Other types of study

	Author, year (Country)	Design	Context	Health professionals involved	Size of the network	Aim	Outcome measures	Key findings
**1**	Dearing et al, 2011 (United States)	Review	Healthcare organisations	N/A review	"The Cancer Research Network (CRN) is a consortium of 14 research centers based in healthcare delivery organizations nationwide" About 45 funded CRN studies are active at any one time.	To describe the concept of an "implementation registry" using the Cancer Research Network as an example	Principles that should guide an implementation registry.	Principles of developing an implementation registry: easily accessible data, trust and asynchronous reciprocity, high expectations and measurable objectives, engagement of the workforce, commitment of lead users, use cognitive science, medical cognition and medical informatics to aid in understanding user experiences, understand that the registry is about the patient as well, quality matters.
**2**	Friberger & Falkman, 2013 (Sweden)	Case study	Swedish oral medicine network	Primarily dentists with an interest in oral medicine	In January 2009, SOMWeb had 120 registered users located at 74 clinics. For the study, 24 participants completed questionnaire, 9 members were interviewed, 10 meetings were observed.	Exploring factors of the VCoP particularly: enablers and challenges, collaboration and outcomes	Online questionnaire, interviews with participants, observation of teleconference meetings. Enablers, challenges, process and outcomes	**Enablers:** having champions and facilitators, collaborating, having experts participating and IT support. **Challenges:** limited time, varying levels of expertise, incorporating evidence into practice. **Processes:** meeting structure and preparations + case submissions discussed. **Outcomes**: professional development (knowledge, learning, identity, enthusiasm and evidence) and clinical impacts (individual cases and treatment models) discussed.
**3**	Galheigo et al, 2019 (Brazil)	Participatory action research	Paediatric occupational therapists	Nine occupational therapy co-researchers, two lead researchers and two occupational therapy students	13	To develop and assess a VCoP	Discussion around specific topics	5 major themes: (i) dialogic engagement, (ii) reflective opportunities, (iii) a sense of belonging, (iv) affirming professional identity, (v) cultivating professional practice knowledge.
**4**	Lewiecki et al, 2019 (United States)	Review	Bone Health	"Physicians of many specialties participated, most commonly endocrinology, rheumatology,	"Over the first 41 months of Bone Health TeleECHO (October 2015 through February2019), 163	The development and applications of the VCoP	The development of Project-ECHO	-Increases knowledge<br>-Lower cost<br>-Convenient<br>-Was of interest to wide range of participants from different medical disciplines

				orthopedics, and primary care. Non-physician participants included advanced practice providers, physical therapists, nutritionists, and exercise physiologists.”	videoconferences were held with a total of 413 in-dividual participants; 192 patient cases were presented and discussed, and 2494 h of no-cost CME were provided.”<br><br>“... Average attendance increased from 15 per session in 2015to 52 in 2018 and continues to rise in 2019. Attendees were located in 42 states in the USA (Fig.1) and 11 other countries (Fig.2).”			-Participants benefited in urban and sub urban locations: isolation not limited to physical location
**5**	Lewis et al, 2011 (United States)	Case study	Social work	Social work educators	Launched in 2009, one year later had 70 members.	To bridge the research to practice gap with a VCoP using Web 2.0	Focus groups and an online survey	-Shows how Web 2.0 can be used<br>-Staff need to be responsive to changing needs.
**6**	Mayrhofer et al, 2015 (United Kingdom)	Discussion paper	Dementia Champions	Health and social care workforce	Dementia symposium: 120 people<br><br>Bi-monthly newsletters to around 180 people	To establish a VCoP and assess the success	Facilitators and barriers - not specifically stated how collected	-Does not conform to models in literature<br>-Also links with NHS trusts, DCs and educators<br>-DCs did not engage with virtual VCoP the university set up.<br>-Needs to become a network within a network<br>-For a VCoP to be successful it needs to be part of an organisation that offers resources.
**7**	Pratte et al, 2018 (Canada)	Mixed-methods design + sequential follow up strategy	Paediatric Physiotherapy	Paediatric physiotherapists	Survey: 41<br>Semi structured interviews: 9	To assess perspectives of the VCoP	Social factors, information quality factors, system quality factors	-Used the “web ergonomics conceptual framework” to interpret results.<br>-Participants were happy with the utility of the VCoP but views on usability were more nuanced

								-Web platforms should be evaluated by usability and utility and designers should take into consideration the needs of participants when designing (also pre-implementation and continual monitoring to make adjustments as needed)
**8**	Rolls et al, 2020 (Australia)	A sequential mixed methods design	Intensive care	All members of the multi-disciplinary team (MDT) although the majority were nurses (84.8%) followed by physicians, industry, academics, healthcare managers and allied health. Large number of intensive care leaders.	2013:1600 (note: contemporary analysis of demographic profiles was not possible post 2009)	To explore the contents of the VCoP	Description of discussion threads, member posting behaviour, knowledge exchanged, analysis of latent content	-Potential for effective VCoP demonstrated<br><br>"The central construct of virtual community work was identified with six elements that facilitated participation and knowledge exchange including: (a) the discussion thread; (b) sharing of artefacts; (c) community; (d) cordiality; (e) maven work; and (f) promotion of the VC"
**9**	Vinson, 2014 (United States)	Integrated mixed methods approach	Cancer control research	Key stakeholders: researchers, healthcare practitioners and intermediaries	Brainstorming: 1500 invited, unable to see how many contributed<br>Sorting: n=39 complete<br>Importance rating: n=57 complete<br>Feasibility rating: n=43 complete	To use concept mapping to develop a framework	Brainstorming, facilitated discussion, rating and sorting of statements, analysis and cluster mapping	Statements grouped in 9- cluster map create framework for this study:<br><br>1. Standardization and best practice<br>2. External validity<br>3. Funding and resources<br>4. Social learning and collaboration<br>5. Cooperation<br>6. Partnerships<br>7. Inclusiveness<br>8. Social determinants and cultural competency<br>9. Preparing the environment
