# Supplementary material for: Implementation of Virtual Communities of Practice in Healthcare to Improve Capability and Capacity: A 10-Year Scoping Review
Source: Int J Environ Res Public Health. 2022 Jun 29;19(13):7994. doi: 10.3390/ijerph19137994 (PMC9265616; doi:10.3390/ijerph19137994)
Supplement: Supplementary file 1 [file ijerph-19-07994-s001.zip › Table S4. Table of VCoP processes.pdf]

Table S4. Table of VCoP processes.

Author(s)	Why was the VCoP developed? What is its purpose?	How did the network develop?	Forms of interaction included in the network?	Theoretical basis/ Framework?
Abos Mendizabal et al (2013)	VCoP HOBE+ developed to foster and facilitate innovation in primary care within the Basque Health System.	Launched in October 2011 by 4 primary districts in the health system and the Basque Institute for Healthcare Innovation.<br><br>Participation is voluntary. All primary care workers in Health System invited to use via email. HOBE + users can share ideas or suggestions for improvements, as well as access those proposed by others. Once ideas have been introduced, users can discuss, enriching them and offering alternatives.	Web based platform only. IdeaScale software used. 4 working groups developed to manage the VCoP:<br>*Ideas Group* -responsible for the initial screening of ideas and galvanisation of the platform.<br>*Innovation Group* - more in-depth analysis, study feasibility of proposals from Ideas Group. Refine proposals and decide whether they could be implemented from this level or need approval from the Management Group.<br>*Management Group* - responsible decisions on proposed changes, improvements or initiatives, as well as integrating them into management plans or passing them onto other levels of management. Identification of corresponding resources and development of innovation projects.<br>*Facilitator role* -coordination of the 3 working groups, preparation and conduction of meetings, and supervision, follow up and support in the development of every selected idea.	States the study confirms the successful factors for VCoPs proposed by Probst and Borzillo and their applicability in the healthcare sector.
Adedoyin (2016)	N/A Rapid review. Aims to synthesise the characteristics of VCoP utilisation across practice settings in social work.	10 included studies for rapid review represent different sectors or domains of social work profession. 4/ 10 studies indicated a rapid rise of the use of VCoPs in social work education. 6/ 10 focused on VCoP utilisation in social service agencies.	N/A Rapid review. All 10 studies hosted on a digital platform.	Part of thematic health VCoP framework (Barnett et al., 2012) +modified versions of 2 previously published systematic reviews combined and used to design coding framework
Alary Gauvreau et al (2019)	Designed to fill gaps in evidence-based practice for speech-language pathologists in aphasia rehabilitation.	13 speech-language pathologists experienced in aphasia rehabilitation in Canada recruited to participate in the CoP. Individual interviews conducted with all participants before the beginning of the CoP, which informed design of the CoP. The CoP was 'top down' and created by the researchers who selected the topics.<br><br>CoP activities developed related to 5 educational aims of knowledge, social interaction, knowledge sharing, knowledge creation and identity. Topics selected considering available evidence, potential for reflective practice, and because they were part of usual clinical activities of speech-language pathologists. Chosen to mirror participant clinical realities and interests.	7 activities offered to CoP members: online meetings about weekly topic; viewing recorded meetings; examination of suggested resources (i.e., clinical tools for goal setting, assessment, and intervention including research articles, websites, and standardized tests); interactive discussion forum about resources; interactive discussion forum related to weekly question; folder to share material used in one's clinical practice; examination of materials shared by others.<br><br>1hour online topic discussions offered 4 times for: goal setting; assessment and out-come evaluation; indirect intervention; discharge.	Theoretical concepts of constructivism, social learning, situated learning, and reflective practice used for design and implementation of the CoP.

			Offline activities available each week. Researchers offered 32 resources for participants to examine and discuss. Question of the week concerned with weekly topic.	
Barnett et al (2012)	Created the health VCoP framework after conducting a literature review of VCoPs in General Practice training using the Probst and Borzillo (2008) framework.	N/A as literature review proposing framework	Developed framework consisting of seven factors:<br>1. Facilitation, 2. Champion and Support, 3. Objectives and Goals, 4. A Broad Church, 5. Supportive environment, 6. Measurement, Benchmarking and Feedback, 7. Technology and Community.	Created the 'Health VCoP Framework' based around Probst and Borzillo's business CoP framework.
Coleman (2012)	To allow doctors from rural hospitals in South Africa to share professional knowledge with specialist doctors at urban hospitals. The paper proposes a VCoP Framework.	Semi structured open-ended interviews conducted with representative doctors from community hospitals in South Africa. Interviews identified various barriers to VCoP development including ICT infrastructure, doctors' workload and their dissatisfaction with poor community of practice. Based on findings proposed a VCoP Framework to support the National Health Insurance project in South Africa.	Proposes a social framework as a guide to the VCoP based on 3 principles of responsibility (doctors needing to take ownership and accountability of the VCoP), connectedness (social elements that engage doctors in VCoP) and reverence (need to respect other users).	Proposed social framework for a VCoP.
Dearing et al (2011)	Case study of the Cancer Research Network (CRN). Explains how an implementation registry could tie together practitioners with researchers.	Principles that should guide planning and development of an implementation registry:<br>1.Organise existing data so that it is easily accessible<br>2.The basis for participation should be trust and asynchronous reciprocity.<br>3.Expectations should be high and objectives measurable<br>4.Engage the workforce.<br>5.Gain the commitment of "lead users" as featured participants<br>6.Use techniques of cognitive science, medical cognition, and medical informatics to understand the user experience.<br>7.An implementation registry is about the patient, too.<br>8.Quality matters	Describes an implementation registry -an online resource for healthcare practitioners that allows for multiple VCoPs facilitated by a common infrastructure. The registry embeds each community in a learning network in which employees of organizations voluntarily unite around a common purpose (e.g., improved transitions of care or medical home models) and interconnect to acquire, organize, and share their knowledge. Could accelerate knowledge sharing, adoption, trial, implementation, and sustained use of innovations	Implementation registry -online resource for healthcare practitioners that allows for multiple VCoPs facilitated by a common infrastructure.
Farrell et al (2014)	Aims to infuse evidence-based strategies into communities by engaging researchers and practitioners in a joint approach to research dissemination.	Cancer Information Service (CIS) Partnership Program evolved from a network into an informal CoP.<br><br>National Cancer Institute (NCI) adapted Wenger's Participatory Framework Model to reflect successful aspects of the CIS Partnership Program and address previously identified gap between cancer control science and practice. NCI focused its work on developing R2R web platform around 3 community dimensions: content, interaction and activity.<br><br>NCI launched series of monthly cyber-seminars focused on translating research to practice, fostering engagement and dialogue by pairing researchers with practitioners.	National Cancer Institute (NCI) launched the website with initial features that included discussions, webinars, featured partners, and events.<br>• Expanded to include R2R Mentorship Program to implement evidence-based programs.<br>• Coalition Corner provides cancer control coalitions a virtual space for asking questions, discussing achievements and sharing challenges.<br>• Virtual cross walk -lead researcher engages community members in how to adapt an intervention.<br>Online discussion platform and archive for the monthly Advanced Topics in Implementation Science webinar series - implementation scientists share perspectives on current topics and their work.	Adapted Wenger's Participatory Framework Model
Friberger & Falkman (2013)	Case study of the Swedish Oral Medicine Network (SOMNet). Members are mainly clinicians and researchers with a special interest in oral medicine. Case consultations	In the early 1990s, four hospital unit clinics began holding telephone conference supported meetings for discussing cases. This evolved into the SOMNet collaboration, which holds monthly telephone conference meetings to discuss difficult and interesting cases. The SOMWeb system was launched in 2006.	SOMNet distance consultation meetings:<br>*Meeting structure:* monthly-one-hour telephone conference meetings, about six cases discussed, led by facilitator.<br>*Case submission main purposes:* seeking advice regarding diagnosis or treatment; wanting to raise a general issue for discussion,	No specific framework. Developed from conferences for discussing cases.

	are focused on generating ideas for diagnosis and treatment, with the goal of improvement and harmonisation of patient care.		educating, and building reputation, participants suggest possible diagnoses and treatments. Research literature important source of external evidence.<br>*Meeting preparations:* participants prepare for meeting by review of case description and images before meetings, performed to aid learning and as a community obligation.	Clinical collaboration with more emphasis on research.
Galheigo et al (2019)	To contribute to the improvement of problem solving and reasoning by occupational therapists in acute pediatric care in resource-challenged settings in Brazil.	The CoP involved 9 occupational therapy (OT) co-researchers, 2 lead researchers and 2 OT students. The co-researchers were recruited from hospitals in Sao Paulo, Brazil through a hospital survey and snowball sampling.<br>The design of the study included a phase during which the invited participants came to understand their roles as co-researchers and co-facilitators of the CoP, while the lead researchers worked as facilitators of the ongoing process.	Ten face to face CoP group meetings over 7 months and a web-based platform where the co-researchers are engaged in virtual activities, such as forums, chats, blogs, surveys, exchange of scholarly literature or other materials. CoP groups were audio-recorded, filmed, and transcribed.<br>Potential topics for discussion were presented, were negotiated with input from participants, and re-confirmed at the end of each meeting.	Participatory Action Research methodology.
Landes et al (2019)	Internal organisation SharePoint network, supporting grassroots implementation of evidence-based practice in areas that do not have access to large scale training and consultation.	Creation of a Veteran Affairs (VA) Dialectical Behaviour Therapy (DBT) VCoP. A SharePoint Web application was created including uploading files and posting in the discussion forum. A champion was appointed for creating and maintaining the VCoP. Categories were created for all the existing materials that were available to post on the site.<br><br>Providers engaged in user acceptance testing of the prototype and gave feedback. Providers helped clarify the objective and goals of the VCoP and were stakeholders willing to support the VCoP.<br><br>Dissemination of the site was via various methods including an email listserv, email signature banners, staff updates, via the Mental Health Services intranet page, training companies for DBT, and conference and training settings.	Facilitation has been engaged in numerous ways to increase and maintain user engagement: new information is continually added to the site (e.g., new handouts, new presentations); VA providers who offer DBT or are DBT experts engaged via e-mail to either add information to the site or to discussion forum posts.<br><br>Second level categories on the website involve sections: an overview of DBT, therapy resources, training and implementation resources, and evaluation and research resources. Another section lists DBT components available across VA medical centres and clinics. A discussion forum allows users to post questions for discussion.<br>2 VA e-mail listservs - a 'news and updates' list and a discussion forum alert e-mail.	Barnett et al's (2012) Health VCoP Framework, plus steps to increase and maintain engagement.
Lewiecki et al (2019)	Bone Health TeleECHO linking healthcare professionals in the treatment of osteoporosis through weekly videoconferences, discussion of clinical questions and a short topic presentation.	Project ECHO began in 2003 and uses videoconferencing technology (mostly Zoom) to create a 'knowledge network' linking specialists in a particular field of medicine. Principal method of learning is through discussion of issues with real but de-identified patients. There is a team dedicated to expanding the global footprint of the TeleECHO model for many disease states, via the creation of ECHO hubs. Project ECHO conduct training to others committed to starting a program.<br><br>The Physical hub is a videoconferencing room with participants logging on from remote locations.	Discussion of clinical questions arising from de-identified patient cases presented by participants. Each ECHO session also includes a short didactic presentation (about 10 to 15 min in length) and discussion, with the topic typically derived from participant feedback or emergence of new guidelines or publications of interest.<br>Case-based discussions generate clinical concepts that may be applicable for many other patients under the care of the teleECHO participants. Presentations are recorded and stored online. A TeleECHO coordinator provides support for logistics.	Project ECHO
Lewis et al (2018)	Dermatology ECHO hub team provides tele-mentoring to primary care providers and provides continuing medical education through case presentations + short didactic presentations. Aim is to	Over the past 2 decades, tele dermatology has been used to bridge the gap between medical expertise and access in the care of skin disease.<br>In 2015, the Department of Dermatology at the University of Missouri (MU Derm), supported by the Missouri Telehealth Network (MTN), implemented the first non-military	The Dermatology ECHO hub team meet with 7 -15 PCPs once a week during an hour-long ECHO session. Each ECHO session consists of 3-5 de-identified case presentations discussed with the Dermatology ECHO hub team, which then gives the diagnosis and recommendations for further management of each case. Each case presentation and subsequent discussion takes 5-15 minutes.	Project ECHO

	learn and adopt best practices in dermatology, to address shortage of dermatologists which may improve patient outcomes and reduce health care costs.	Dermatology ECHO in the United States as part of a larger ECHO implementation, Show-Me ECHO. The aim was to facilitate management of various dermatological conditions by primary care physicians (PCPs) at distant sites across Missouri.	Each ECHO session also consists of a brief 10-minute continuing medical education (CME)-approved didactic presentation.	
Lewis et al (2011)	Launched in 2009, describes a VCoP model developed using an online social networking service for the University of Buffalo's School of Social Work to bridge the divide between academic research and practice and promote best practice in community agencies where students complete social work practicum hours.	First step was identification of project goals and aims. Focus groups and a survey were completed by social work educators. Results indicated an interest in networking around issues in the profession, specific topic areas and best practices in the field. The problem was matched to technology. A free social networking site was used to develop a platform through which individuals could share information. The School of Social Work's Field Education social networking site was launched in 2009 and 1 year later there were over 70 members. The group is small and actively coalescing, moving toward the active stage of development described by Wenger.	Free social networking site to develop a platform through which individuals could share information. Participants play a role in problem-solving shared experiences and provide resources and referrals. Additionally, school faculty has used the site to recruit research sites, and to notify practitioners of program evaluation opportunities. Options for privacy controls were deemed essential. Focus groups were also conducted as well as an online survey.	Wenger's developmental stages of CoP
Mayrhofer et al (2015)	DEMCoP project sought to create infrastructures of specialist support and shared learning.	Project activities began by hosting an inaugural Dementia Champions Symposium in partnership with the NHS in Hertfordshire and the Alzheimer's Society. Subsequent meetings held with representatives from different NHS primary, secondary and mental health provider units. Dedicated 'Dementia Champions' online-site provided on the University of Hertfordshire server, which required DCs to register as a member.	Hosted an inaugural Dementia Symposium in partnership with the NHS in Hertfordshire and the Alzheimer's Society, which was attended by 120 health-care professionals from across the county.<br><br>Online resource that brings together best evidence and web links to other resources, organised by topic and featuring videos of DCs talking about recent innovations. Videos of DCs talking about recent innovations. Creation of option for discussion groups. Supplemented by a bi-monthly newsletter, meetings, survey and discussions.	No framework reported. Approach sought to be bottom up and context sensitive.
McLoughlin et al (2018)	Integrative review to determine the application of VCoPs for interprofessional education (IPE) and interprofessional collaboration (IPC).	N/A Integrative review	Describes different technological infrastructures that are used for VCoPs and the advantages and disadvantages of these including social networking sites (e.g., Facebook, Twitter, LinkedIn) and online discussion forums, groups or electronic boards. McLoughlin et al conclude that VCoPs have a key role in the future of IPE and IPC by providing the ability to overcome the hurdles of more traditional forms of communication and education.<br><br>For VCoPs to be successful, issues of privacy, trust, encouragement and technology need to be addressed.	No framework reported
Murad et al (2019)	A Community Fracture Capture (CFC) Learning Hub (an interactive, case-based, Web-based learning tool) was designed to help GPs improve the care of patients with osteoporosis and mitigate the barriers to participation in Web-based learning. The paper quantifies GPs' engagement in the VCoP to demonstrate a novel design for	Project team comprised a mix of specialists in the field: experienced GPs; information systems researchers; technology experts; a project coordinator; and specialist physicians with expertise in bone health and osteoporosis.<br><br>The lead time to the commencement of the CFC Learning Hub was 2.2 years. On average the project lead spent approximately 3 hours/ week during the lead time to the project launch and 1.5hours per week during the active phase of the project.	The CFC Learning Hub platform had 2 resources for GP interactions:<br>1.A Web-based knowledge repository (the Knowledge Hub) containing curated and prepopulated evidence-based research articles and other resources.<br>2.An online social network forum (the Discussion Forum) where GPs could freely post online comments, including:<br>questions for discussion posted by facilitators, case studies to encourage GPs to learn and share their knowledge based on shared experiences and relevance to their immediate clinical	No framework reported

	continuing professional development.		practice; topical discussions as either (a) hot topics (HT) deemed relevant for GPs, posted by our osteoporosis specialists or (b) other topics (OT) that were open for wider discussion (i.e., introductions, where facilitators and GPs introduce themselves, and burning questions, where GPs and facilitators post inquiries on osteoporosis.	
Pratte et al (2018)	A 5-month VCoP developed to improve the implementation of pediatric physiotherapist (PT) best practices in the management of children with developmental coordination disorder (DCD).	The VCoP was developed and launched after a need was expressed and identified by pediatric physiotherapists working with children with DCD in Quebec, Canada. PTs were recruited after a face-to-face workshop on the management of DCD in collaboration with a pediatric rehabilitation centre that provides continuing education. Recruited PTs were granted access to the web platform, and they could then create an online profile. 4 PTs with expertise in DCD were the facilitators of discussions, referred users to information and helped qualify the level of scientific evidence of the content on the platform.	VCoP web platform was divided into 7subsections: 5 subsections from the themes presented in the online module on developmental coordination disorder, and 2 subsections on news and other topics. PTs were invited to post questions, respond to questions posted by colleagues, and/or share resources by attaching documents to posts.<br><br>3 features: Traffic light color coding to indicate the level of evidence supporting a post, a 'thumbs up' feature to indicate when a post was of particular interest, a search function allowing users to find specific posts. For PTs to keep track of VCoP activities, there were also (1) monthly emails summarizing recent VCoP activity, (2) the possibility to subscribe to email notifications for new content.	No specific framework to develop. Platform based around literature on factors influencing quality of VCoPs - social factors, information quality factors, and system quality factors.
Ranmuthugala et al (2011)	Systematic review on how and why CoPs are established in healthcare and whether they improve performance of healthcare organisations.	How the networks developed was not specifically described as part of this review.	16 of the 31 primary research studies included in the review included face-to face CoPs. Email and web-based systems were the next most popular method. Nil else stated.	N/A literature review. Though states it is 'important to know how best to, as suggested by Wenger and colleagues, cultivate CoPs to benefit their organisations.'
Rolls et al (2020)	ICUConnect is a listserv created to facilitate communication and knowledge sharing between clinicians of the adult ICUs in NSW, Australia.	Not stated.	VCoP was an intensive care listserv (2003 -2013) to facilitate communication and knowledge sharing between clinicians of 43 adult ICUs in NSW, Australia. The most direct action was posting within a discussion thread with 3 main sub-elements: request for and supply of knowledge, purpose and concerns that motivated member to post. Purpose for all first posts was to 'benchmark' practice. 6 elements of virtual community identified: the discussion thread, sharing of artefacts, community, cordiality, maven work, and promotion of the VC.	States 'the study was underpinned by pragmatism (James, 1907–2013) and informed by the theories of community of practice (CoP; Wenger, 2004) and diffusion of innovations (Rogers, 2003).'
Rolls et al (2016)	An integrative literature review (1990 – 2015) on the use of social	36 reports described 31 VCoPs established in 3 main ways: discussion forums or Listservs created by a professional society, open access platforms such as Yahoo or Twitter, or established by	Common types of social media platforms identified were Listservs (n=22), Twitter (n=18), general social media (n=17), discussion forums (n=7), Web 2.0	N/A literature review

	media by health care professionals in developing VCoPs.	a government health department to improve communication and knowledge distribution between health care professionals to enhance care.	(n=3), topic-specific discussion forums plus document repositories (n=3), a wiki (n=1), and Facebook (n=1).	
Sims (2018)	Members of the case organisation are specialised medicine doctors working in complete or semi-isolation in very remote locations, where challenges arise often, and support is not easily available.	Not stated.	The community structure is built around a blog and an email list. Interaction takes place by blog postings, comments on the blog postings, and emails between members.	Specific framework not stated. Discusses theoretical framework. Reports CoPs based on trust, perceived usefulness, knowledge and support, simple forms of computer-mediated support, organisations open to change.
Ting et al (2018)	CanadiEM is a national website to foster a virtual community of practice that facilitates sustainability, collaboration, and education scholarship while developing junior digital scholars.	CanadiEM was formed in 2016 from the merger of two Canadian websites and a podcast.<br><br>Using a community-of-practice model, two training programs were introduced to support junior community members in becoming core editorial team members and employed asynchronous Web technologies to facilitate collaboration. A coached peer review process was also introduced and formed strategic alliances that aim to ensure a high quality of publication.	CanadiEM accepts article submissions openly through a publicly available e-mail address, allowing peripheral members to contribute and further the goals of the community.<br><br>Content experts assist 2 programs: CanadiEM introduced a coached peer review process, and creation of strategic alliances with nationally recognized organizations, including the Canadian Journal of Emergency Medicine (CJEM), the Canadian Association of Emergency Physicians (CAEP), and Translating Emergency Knowledge for Kids (TREKK). These organizations provide educational resources for dissemination via the online platform.<br>5 primary content sections: medical concepts, podcasts, academics, opinion & arts and subsections.	Organizational chart based on Lave and Wenger's Community of Practice.
Vinson (2014)	Concept mapping to develop a conceptual framework for creating VCoPs for translate of cancer research into practice.	No actual VCoP analysed. Used concept mapping and an integrated mixed-methods approach to examine the issues key stakeholders believed needed to be addressed to create and sustain government-sponsored virtual communities of practice to integrate cancer control research, practice and policy.<br>Phase 1: Brainstorm facilitation of research into practice. Following there was facilitated discussion with advisory group of experts to use key word grouping for statements. 193 statements reduced to 70 statements used for phase 2.<br>Phase 2: Sorting the statements into conceptual groups + rating statements on importance and feasibility.<br>Final phase: Members of the Virtual Communities of Practice working group help interpret the results of the project and helped select a cluster map that was best fit for the respondent data.	Developed point cluster map depicting a conceptual framework for creating VCoPs for moving cancer control research into practice. Framework includes:<br>1. Standardization and best practices<br>2. External validity<br>3. Funding and resources<br>4. Social learning and collaboration<br>5. Cooperation<br>6. Partnerships<br>7. Inclusiveness<br>8. Social determinants and cultural competency<br>9. Preparing the environment	Developed cluster map of best conceptual framework for VCoP.

Wolbrink et al (2017)	OPENPediatrics is an online social learning platform developed at Boston Children's Hospital in collaboration with the World Federation of Pediatric Intensive and Critical Care Societies and the IBM Corporation. The strategy aimed to engage and coalesce the global community of critical care clinicians.	A worldwide needs assessment was conducted prior to developing OPENPediatrics, with respondents requesting question-and-answer forum, best practice webinars, and a place to network and talk with doctors and nurses worldwide. An asynchronous format was chosen.<br><br>The World Shared Practices video (WSP) series was launched in March 2013 to engage the global community of critical care clinicians.	Monthly, a well-known, international expert in pediatric critical care medicine is a speaker for the WSP series. WSPs are filmed during their visit to Boston Children's Hospital, and videos edited and reviewed. Each 30-45min video contains 2 to 5 questions on context and practice, with expert opinion later provided. Viewers leave comments that display alongside the video.<br>WSPs can be viewed whenever it is convenient for the viewer. Clinicians are encouraged to hold an educational conference at their hospitals to watch the videos together, discuss responses to the questions, and submit a single group comment.	World Shared Practices. Asynchronous format and based on results of a needs assessment.
