# Supplementary material for: Implementation of Virtual Communities of Practice in Healthcare to Improve Capability and Capacity: A 10-Year Scoping Review
Source: Int J Environ Res Public Health. 2022 Jun 29;19(13):7994. doi: 10.3390/ijerph19137994 (PMC9265616; doi:10.3390/ijerph19137994)
Supplement: Supplementary file 1 [file ijerph-19-07994-s001.zip › Table S5_Table of VCoP roles.pdf]

Table S5. Table of VCoP roles.

Study ID	*Sponsor / key organisation*	*Facilitators/ leaders*	*Expertise or other roles?*	*Members*
AbosMendizabal et al (2013)	Launched by 4 primary districts of Osakidetza and the Basque Institute for Healthcare Innovation (O + berri). O+berri plays the coordinator role between Osakidetza Headquarters and HOBE+ members.	Facilitator role includes the coordination of the 3 working groups, preparation and conduction of the meetings of each group and supervision, follow up and support in the development of ideas.	Ideas Group responsible for initial screening; Innovation group carries out more in-depth analysis and studies feasibility of proposals; Management Group has ultimate responsibility for taking decisions on proposed changes, improvements or initiatives.	All primary care workers from Biscay and Araba invited.
Adedoyin (2016)	Not stated -literature review			
Alary Gauvreau et al (2019)	Study was undertaken as part of first author's PhD. CoP was created by the researchers who chose the topics.	First author (CAG) was the facilitator.;	GLD (co-author), a professor in speech-language pathology, participated in the design of the activities and provided expertise about resources in participation-based aphasia rehabilitation. A research assistance took care of technical aspects of the online meetings.	14 speech-language pathologists with experience in aphasia rehabilitation.
Barnett et al (2012)	Literature review. Reports in health the agenda is usually driven by the organisation attempting to start the community or the researchers -someone had to start the network then continue to support its activities. Ongoing organisational support essential to success of projects. Leader can be the initiator of the community, in many studies, this role can be performed by the study organisers.	Most common role described in the studies was of facilitator or moderator. The originator of the group may end up being the initial leader and facilitator. The facilitator may be appointed by the originators of the group, or the facilitators of the group may arise spontaneously. Leaders can emerge in the absence of facilitators. The role of leader and facilitator may be separated or performed by the same person. Main importance of the leader in this review is in the initiation of the community.	Moderators and active group continue to stimulate and promote knowledge sharing. In almost every study, there were either a variety of practitioner types or a variety of organisations participating.	Core group consists of a minority of active users, whilst often the majority is passive.
Coleman (2012)	10 community hospitals in the North West Province of South Africa who communicate with urban hospitals to support the National Health Insurance Project.	VCoP does not exist yet. Paper proposed a social framework for a VCoP. Paper suggests Doctors themselves need to take ownership and accountability of the VCoP.		A doctor representative from each of the community hospitals.
Dearing et al (2011)	Provides example of Cancer Research Network -consortium of 14 research centres supported by the National Cancer Institute. Though suggests principles for an implementation registry, e.g., a trans-institutional network.	CRN includes a Principal Investigator's Office, Executive and Steering Committees, Academic Liaison Committee, and Administrative Committees.		CRN researchers across healthcare organisations -each existing in several communities. In suggested implementation registry, employees such as nurses and physicians to be engaged.
Farrell et al (2014)	National Cancer Institute created online CoP 'Research to Reality'	Not stated other than National Cancer Institute		Researchers and practitioners.

Friberger & Falkman (2013)	4 hospital unit clinics began holding telephone conferences which evolved into the Swedish Oral Medicine Network.	Monthly meetings led by chairperson who facilitates the monthly meetings and reviews the cases to be presented. Meeting's facilitation rotates among core members.	The collaboration's founder has acted as a champion, through initial chairing of meetings, organisational and technical development, and outreach to potential members. Most speakers at meetings are experts. The collaboration includes several oral medicine experts. The core group consists of members that take on leadership roles and set the agenda for the group.	Clinicians (dentists) and researchers with a special interest in oral medicine. The active group's members are regular participants in the community's events and sometimes participate in discussions, but without the intensity of the core group. A large portion of the participants often belongs to the group of peripheral users, who mostly observe interactions between core and active members.
Galheigo et al (2019)	Participatory Action Research model. Members recruited by lead researchers from hospitals in the city of Sao Paulo, Brazil.	Participants also had roles as co-researchers and co-facilitators of the CoP, while the lead researchers worked as facilitators of the ongoing process.	A guest discussant, a seasoned clinician and researcher was an occasional member who took part in a few activities and was invited to bring more input to the discussions. Two research collaborators with international scholarly expertise in the field were also invited to consult on the project as it unfolded.	9 occupational therapy co-researchers, 2 lead researchers, 2 occupational therapy students (who provided practical and technical support).
Landes et al (2019)	First author, who worked for Veterans Affairs, created the website and categories for material.	Facilitation engaged in numerous ways to increase and maintain user engagement -site created with input and engagement from VA DBT community, VA providers who offer DBT or are DBT experts add information or reply to discussion forum posts. Email listserv used to alert of discussion forum posts.	Providers across the VA who were providing DBT.	Any VA employees interested in DBT can participate or use the VCoP.
Lewiecki et al (2019)	Project ECHO	Funding for 10hrs of staff time per week, IT support, and continuing medical education is provided by Project ECHO. A teleECHO coordinator provides support for logistics.	Project ECHO provides training for anyone who wants to learn about the model of learning. YouTube videos provide information on aspects of Bone Health TeleECHO. Collaborative videoconference is held every 3 months.	Healthcare professionals of many disciplines-both physicians and non-physicians.
Lewis et al (2011)	Partnership between the University of Buffalo School of Social Work and the University of Buffalo Teaching and Learning Centre.	Not specifically stated other than 'project staff'		Social workers and social work educators.
Lewis et al (2018)	Project ECHO. The Department of Dermatology at the University of Missouri leader in teledermatology. Supported by the Missouri Telehealth Network (MTN) implemented Show-Me ECHO.	The MTN provides operational, technical, and recruitment support.	The Dermatology ECHO hub team consists of 7 providers, including physicians, a psychologist, and an advanced practice nurse.	Rural primary care providers in Missouri, USA.
Mayrhofer et al (2015)	Health Education England commissioned a project to develop and maintain a system wide initiative to support Dementia Champions in Hertfordshire. The project was based at the University of Hertfordshire, who provided a dedicated online site.	Inaugural Dementia Champions Symposium held in partnership with NHS Hertfordshire and the Alzheimer's Society. Meetings held with representatives from different NHS primary, secondary and mental health provider units.	Clinical leads in NHS Trusts.	Dementia champions -health care professionals and key clinicians.

McLoughlin et al (2018)	Not reported -literature review	Review states leaders or facilitators can play an important role in encouraging activity participation, and in establishing and maintaining successful communities.		Several articles reported participation issues. A large proportion of members function in the role of 'lurkers' (non-active participants). Active participation of all members is important and should be encouraged.
Murad et al (2019)	Project team from Melbourne consisting of mix of specialists in the field: experienced GPs, information systems researchers, technology experts, a project coordinator, and specialist physicians with expertise in bone health and osteoporosis.	A facilitation team, formed to incentivize the engagement of the participants and facilitate discussion in the online discussion forum, included 4 specialist physicians, 2 senior GPs as facilitators, 1 dedicated content facilitator, and 1 information technology administrator.		7 General Practitioners.
Pratte et al (2018)	Research team		4 Physio's with expertise in DCD were identified as knowledge brokers -initiated discussions, referred colleagues to existing information and helped qualify the scientific information on the site. 3 clinical experts were invited to lead week-long discussions.	Paediatric physiotherapists managing children with developmental coordination disorder.
Ranmuthugala et al (2011)	N/A literature review	Not stated.		
Rolls et al (2016)	N/A literature review			
Rolls et al (2020)	Large open VC, multi-organisational. Created to facilitate communication and knowledge sharing between ICU clinicians. No specific sponsor/ key organisation stated.	2 authors (intensive care nurses with extensive clinical experience) members of the VC with one moderator 2005-2014, including managing member enrolment and ensuring discussions adhered to etiquette.	High rate of intensive care leader participation. Knowledge contributed by clinical leaders from 80 facilities throughout Australasia. Collective knowledge creation through VCoP members.	Clinicians of the 43 adult ICUs in NSW, Australia. All health care professionals included though majority were nurses. Large numbers of intensive care leaders.
Sims (2018)	Membership voluntary and elective.	Not stated -'self-selecting members		Specialised medicine doctors working in complete or semi-isolation
Ting et al (2019)	Formed from merger of 2 Canadian websites and a podcast 'Free Open-Access Medical Education'. Founded by nationally acclaimed medical educators.	Editorial Boards consists of 2 editors-in-chief (experienced graduate-trained clinician educators) who oversee 16 senior editors and 40 junior editors. Rest of initial editorial board consisted of digital mentees.	Core members have two opportunities for apprenticeship: the Junior Editor program and the Digital Scholars program. Supervisors facilitate access to international leaders in medical education. CanadiEM includes a coached peer review process connecting trainee authors with content experts. Also, strategic alliances with nationally recognised organisations.	Online audience comprises peripheral membership.
Vinson (2014)	N/A -uses concept mapping to examine issues need to create government-sponsored VCoPs for cancer research, practice and quality.		Cooperation cluster focuses on engaging broad and diverse expertise and organisations in government sponsored VCoPs.	Participants must have the ability and desire to collaborate. Participants need to be able to share information about their work and need their organisation support to allow this sharing to happen. Types of participants that should be included include health care providers, office managers, community organisations, health departments, federally qualified health centres, survivors, family members.

Wolbrink et al (2017)	OPENPediatrics (an online social learning platform) launched the World Shared Practices video series to engage and coalesce the global community of critical care clinicians.	Each month, a well-known international expert in pediatric critical care medicine is invited to be a speaker for WSP, by 4 faculty members in pediatric critical care medicine at Boston Children's Hospital.		Critical care clinicians globally.
